# Remnant cholesterol and associations with incidence, progression, and change in coronary artery calcium in South Asian Americans

**DOI:** 10.1016/j.jacl.2025.09.008

**Published:** 2025-10-28

**Authors:** Eve M. Manghis, Meghana D. Gadgil, Matthew Budoff, Namratha R. Kandula, Alka M. Kanaya

**Affiliations:** Division of General Internal Medicine, Department of Medicine, University of California, San Francisco, CA, USA; Division of General Internal Medicine, Department of Medicine, University of California, San Francisco, CA, USA; Division of Cardiology, Harbor-UCLA and The Lundquist Institute, Torrance, CA, USA; Department of Medicine and Preventative Medicine, Northwestern University Feinberg School of Medicine, Chicago, IL, USA; Division of General Internal Medicine, Department of Medicine, University of California, San Francisco, CA, USA

**Keywords:** Remnant cholesterol, Coronary artery calcium, Atherosclerosis, Risk factors, Dyslipidemia

## Abstract

**BACKGROUND:**

Remnant cholesterol (RC) may contribute to residual risk of atherosclerotic cardiovascular disease (ASCVD), but few studies have examined this in South Asian populations, who experience a disproportionately high incidence of ASCVD.

**OBJECTIVE:**

Using data from the Mediators of Atherosclerosis in South Asians Living in America (MASALA) Study, a community-based longitudinal cohort of South Asian men and women without ASCVD at baseline, we examined the association between RC and change in coronary artery calcium (CAC).

**METHODS:**

Baseline fasting blood specimens and questionnaires were obtained between 2010–2013. CAC was assessed both at baseline and after 5-year follow-up in 698 participants. We used multivariable linear and logistic regression to examine relationships between RC quartile and CAC incidence (no CAC at baseline to any CAC at examination 2), CAC progression, and overall change in CAC.

**RESULTS:**

In 892 MASALA study participants at baseline, mean RC was 25.8 ± 11.8 mg/dL. Incident CAC occurred in 25.8% of participants and increased across RC quartiles ( *P* = .002). After covariate adjustment, individuals with RC levels in the third (odds ratio [OR]: 2.24, 95% CI: 1.01–4.98) and fourth quartiles (OR: 2.57, 95% CI: 1.20–5.51) had significantly higher odds of incident CAC after 5 years than those in the lowest RC quartile. These associations persisted after sequential adjustment for behaviors, adiposity, and measures of insulin sensitivity and did not vary by gender. RC quartiles were not associated with CAC progression or CAC change.

**CONCLUSION:**

Higher RC levels were associated with increased odds of incident CAC independent of risk factors for ASCVD.

## Introduction

South Asian populations have a disproportionately high burden of atherosclerotic cardiovascular disease (ASCVD). ^1^ Compared to individuals with heritage from other racial and ethnic groups, South Asians have a higher risk of incident ASCVD, develop ASCVD earlier in life, and have higher rates of triple-vessel coronary artery disease, even at lower levels of low-density lipoprotein (LDL)-cholesterol. ^2–4^ LDL-lowering pharmacotherapy has long been the cornerstone of atherosclerotic disease prevention and management. ^5^ However, even after optimal lowering of LDL, ASCVD risk still persists. ^6–8^ Furthermore, existing Pooled Cohort Equations may underpredict ASCVD in South Asians, underscoring a clear need to explore new markers of risk. ^9^

Remnant cholesterol (RC) is a cholesterol contained in triglyceride-rich lipoproteins after triglycerides have been removed, and RC has been identified as a potential contributor to this risk. ^10^ RC is primarily composed of very low-density lipoproteins (VLDL) and intermediate-density lipoproteins (IDL) and can either be directly measured or calculated as total cholesterol minus LDL-cholesterol minus high-density lipoprotein (HDL)-cholesterol. ^10^ RC promotes vascular calcification via both endothelial dysfunction and inflammation. ^11, 12^ Early studies on RC demonstrated that in individuals with high baseline cardiovascular risk, elevated RC increased the risk of developing adverse cardiovascular outcomes. ^13, 14^ More recent data have demonstrated that elevated RC levels are associated with incident ASCVD independent of traditional risk factors, LDL, and apolipoprotein (apo)B levels. ^5^

Although RC has been associated with ASCVD incidence and progression in large population-based cohorts, South Asian populations have not been well-represented in these studies. Further research is needed to assess whether RC is independently associated with the presence of baseline coronary atherosclerosis and progression over time, and whether this association is independent of traditional and other cardiometabolic risk factors. We aimed to assess the association of RC and changes in coronary artery calcium (CAC) score as a marker of subclinical atherosclerosis in South Asian middle- and older-aged adults. Understanding this relationship may inform improved risk prediction and tailored interventions to reduce ASCVD burden in this disproportionately affected population.

## Methods

### Study population

We examined data on South Asian American participants aged 40 to 84 years without prior cardiovascular disease who participated in the Mediators of Atherosclerosis in South Asians Living in America (MASALA) study, a community-based longitudinal cohort of South Asian men and women in the San Francisco Bay Area and greater Chicago areas. Study methods have been published previously. ^15^ Institutional review board committees at Northwestern University and the University of California, San Francisco approved study protocols and all participants gave written informed consent.

Briefly, 2 MASALA field centers recruited and enrolled 906 participants from October 2010 through March 2013. During baseline examination, participants completed questionnaire items including data pertaining to demographic information, socioeconomic status, acculturation, tobacco and alcohol use, medical history, physical activity, diet, and psychosocial measures. ^15^ Trained bilingual research staff recorded participant weight, height, and measured seated blood pressure using standardized protocols.

### Primary predictor

We defined RC as total cholesterol minus LDL-cholesterol minus HDL-cholesterol as described in prior reports. ^16–18^ Of 906 participants enrolled at baseline, a total of 893 individuals had values for RC. We excluded 1 participant from analysis who had a recorded RC of −16 due to clinical implausibility.

### Outcome

Participants underwent a noncontrast cardiac computed tomography (CT) and a single slice abdominal CT at baseline. The CT protocol and interpretation for MASALA have previously been reported. ^19^ Participants underwent cardiac-gated CT scans in either San Francisco or Chicago. Radiologists at the central reading center at Lundquist-UCLA reported coronary calcium score using the Agatston method. Participants were invited back approximately 5 years later at examination 2, which occurred from September 2015 to March 2018, with 83% retention of participants overall, and a total of 701 participants had CAC scores from both examination 1 and examination 2.

We utilized definitions of incident CAC as reported previously, any CAC at examination 2 in a participant who had no CAC at examination 1. ^19^ We defined CAC progression as increase in CAC among those with established CAC > 0 at examination 1. A third variable, CAC change, utilized data from all participants, including those with CAC of 0 at baseline, and was defined as the difference in CAC score between examination 1 and examination 2.

### Covariates

Body mass index was calculated as weight (in kg) divided by the square of height (in meters). Hypertension was classified for any participant with an average systolic blood pressure ≥140 mm Hg or diastolic blood pressure ≥90 mm Hg, or if the participant was taking an antihypertensive medication. Smoking behavior (cigarette use: current, former, never) and alcohol intake (drinks/week) were determined by questionnaires. Fasting blood was drawn to measure fasting plasma glucose, glycated hemoglobin (HbA1c), triglycerides, total cholesterol, and HDL-cholesterol (LDL-cholesterol concentration was calculated). The homeostasis model assessment of insulin resistance (HOMA-IR) was used as a measure of insulin resistance and calculated as insulin (mIU/mL) × glucose (mmol/L)/22.5. ^20^ Adiponectin, an anti-inflammatory protein made by adipocytes, was measured using Millipore Luminex adipokine panel A (EMD Millipore, Billerica, MA) with an interassay coefficient of variation of 2.3% to 4.1%. ^21^

Measures of hepatic attenuation, ectopic fat, and pericardial fat volume were obtained with noncontrast CT images at baseline with electron-beam or multidetector CT scanners. The entire heart and 45 mm of adipose tissue around the proximal coronary arteries were included in CT imaging. Lower levels of hepatic attenuation in Hounsfield Units (HU) demonstrate higher degrees of liver fat. We defined fatty liver as a dichotomous variable with hepatic fat attenuation < 40 HU. ^22^

There were a total of 701 participants who had CAC scores from both examination 1 and examination 2. We excluded 3 participants with prevalent cardiovascular disease at examination 1. A total of 693 participants had measured levels of RC and CAC scores from examination 1 and examination 2.

### Statistical analysis

We categorized RC into quartiles and compared demographic, behavioral, clinical, and metabolic characteristics across the 4 quartiles with analysis of variance, chi-squared tests, or Kruskall-Wallis tests as appropriate.

We analyzed the distribution of incident CAC, CAC progression, and CAC change by RC quartile. Unadjusted analyses used chi-squared for incident CAC and Kruskal-Wallis tests to derive *P*-values. We additionally adjusted for age, gender, diabetes, smoking status, hypertension, and cholesterol medication use for incident CAC.

We created linear and logistic regression models for univariate analyses and multivariable models to examine relationships between incident CAC, CAC progression, and CAC change. We used linear regression models for CAC progression and CAC change for these continuous outcomes and logistic regression models for incident CAC which was binomial. We compared sequential models for each outcome beginning with a base model adjusted for age and gender only, followed by variables selected based on traditional ASCVD risk equations (hypertension, diabetes, smoking, and cholesterol-lowering medications used). Next, we added health-related behaviors (exercise, alcohol use), then measures of adiposity (visceral fat area, liver fat attenuation, and pericardial fat volume), and finally measures of insulin signaling (HOMA-IR, homeostasis model assessment of beta-cell function [HOMA-B], and adiponectin). ^23, 24^ We utilized a backwards selection process, retaining variables associated with *P* < .10 in subsequent models and excluding those without statistical significance.

We used STATA 18.0 and SAS 9.4 for all statistical analyses.

## Results

In 892 MASALA participants from examination 1 without cardiovascular disease at baseline, mean age was 55.3 ± 9.4 years, 53.4% were male, and 27.2% were using a statin. Mean RC was 25.8 ± 11.8 mg/dL and did not vary by gender. Characteristics of the MASALA population at examination 1 are shown in Table 1, stratified by RC quartile. Participants with RC in the fourth quartile (mean RC 41.0 ± 10.3 mg/dL, range 31.0–96.0 mg/dL) were more likely to be male, smoke tobacco, be less physically active, have higher body mass index (BMI) and other adiposity measures (including pericardial fat volume, visceral fat area, and hepatic fat), higher levels of HOMA-IR, HOMA-B, and fasting insulin, and higher prevalence of diabetes ( *P* < .05).

**Figure 1.**
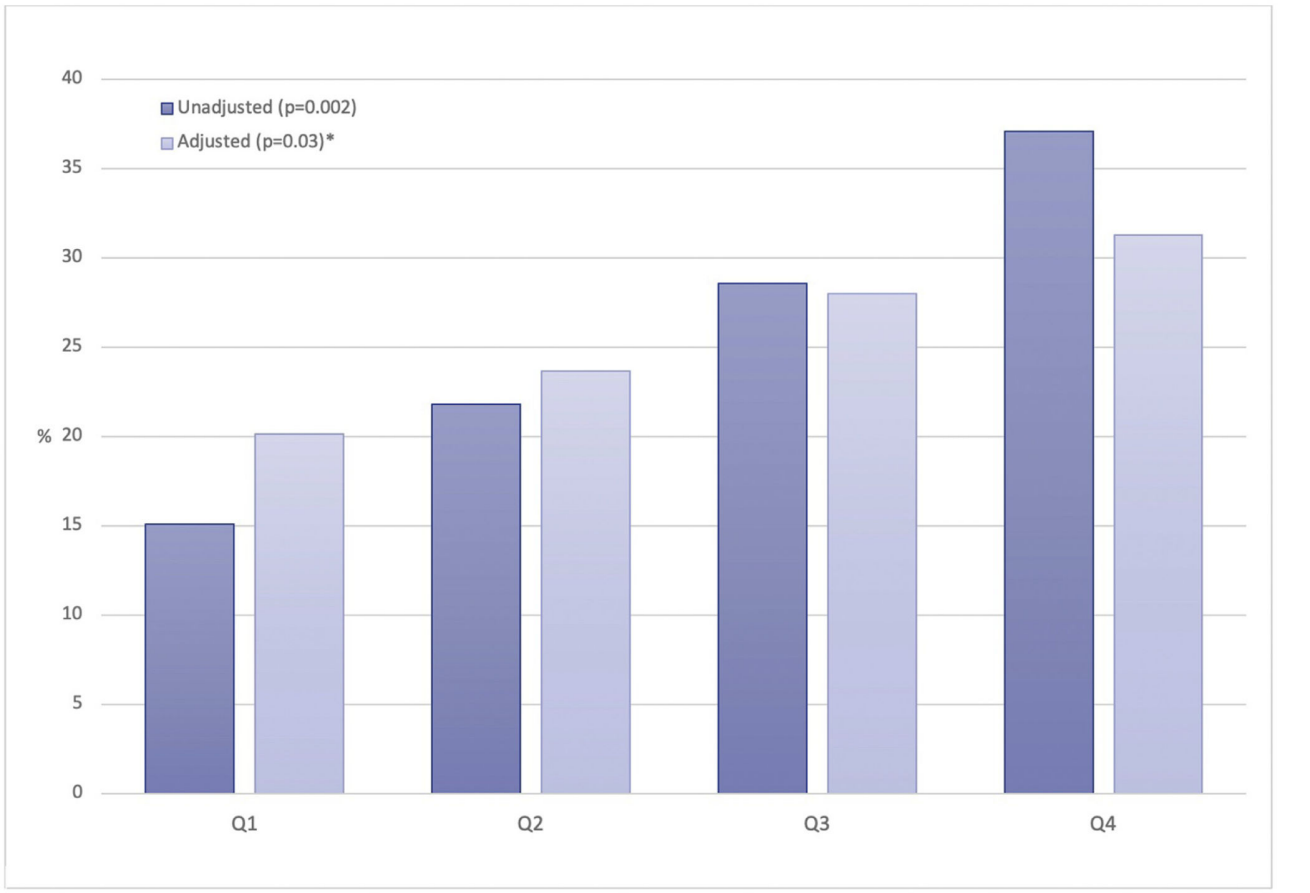
Unadjusted and adjusted percentage of incident CAC among MASALA participants with no evidence of CAC at baseline, comparing by RC quartile. *Adjusted for age, gender, diabetes, smoking status, hypertension, and cholesterol medication use. Abbreviations: CAC, coronary artery calcium; MASALA, Mediators of Atherosclerosis in South Asians Living in America; RC, remnant cholesterol.

There were 698 MASALA study participants who underwent repeat CT scan at examination 2 after mean follow-up time of 4.8 ± 0.8 years. Among the 430 participants with CAC = 0 at baseline, 25.8% developed incident CAC at follow-up. The proportion of participants with incident CAC increased progressively across quartiles of baseline RC, from 15.1% in the lowest quartile to 37.1% in the highest quartile (*P* = .002, Fig 1 ). This association persisted after adjustment for age, gender, diabetes, smoking status, hypertension, and use of cholesterol-lowering medications (*P* = .003, Fig 1 ). There was no difference in CAC progression or CAC change by RC quartiles.

In all multivariable models, RC levels in the third and fourth quartiles had significantly higher odds of incident CAC at 5 years than those in the lowest quartile of RC ( Table 2 ). After adjusting for age, gender, smoking status, hypertension, diabetes, and cholesterol-lowering medication use, odds of incident CAC at 5 years was 2.24 (95% CI: 1.01–4.98) in the third quartile and 2.57 (95% CI: 1.20–5.51) in the fourth quartile ( *P* < .05). This association did not vary by gender. In subsequent models adjusting for these baseline factors as well as exercise and alcohol use, odds of incident CAC in the third (odds ratio [OR] 2.27; 95% CI: 1.02–5.07) and fourth (OR 2.57; 95% CI: 1.19–5.53) RC quartiles were again persistently higher than those in the first quartile. Similar results were found after adjusting for ectopic abdominal fat measures and BMI, and again after adjusting for HOMA-IR, HOMA-B, and adiponectin. In a final, fully-adjusted multivariable model retaining all significant (*P* < .10) variables, odds of incident CAC in RC quartiles 3 (OR 2.55, 95% CI: 1.09–5.96) and 4 (OR 2.85, 95% CI: 1.24–5.57) remained significantly higher than those in quartile 1.

## Discussion

In the MASALA study population, higher than median RC was associated with greater odds of incident CAC, independent of traditional ASCVD risk factors, and even after adjusting for behavioral measures, adiposity measures, and advanced cardiometabolic biomarkers. These findings support RC as a potential target for novel therapies to lower ASCVD risk.

The findings that RC is independently associated with incident CAC is concordant with prior literature. In a large cohort of individuals free of cardiovascular disease at baseline, Quispe et al. found that elevated RC levels were associated with ASCVD independent of traditional risk factors, with highest risk in individuals with high RC and low LDL-cholesterol. ^5^ Hao et al. evaluated CAC progression over median follow-up of 8.6 years using data from the Multi-Ethnic Study of Atherosclerosis (MESA) and the Coronary Artery Risk Development in Young Adults (CARDIA) study cohorts. In this study, 42.5% of participants had CAC progression, with increasing risk of progression in higher RC quartiles and the strongest effect in those without detectable CAC at baseline. ^10^ Mean RC in this sample was notably lower (21.2 ± 8.0 mg/dL) than in the MASALA population (25.8 ± 11.8 mg/dL).

Aside from our study, Hao et al. is the only other known analysis utilizing CAC as a measure of subclinical atherosclerosis, which may identify early coronary disease that can be intervened upon prior to development of symptoms. In contrast, many prior studies examining RC as a predictor variable have relied on angiographic stenosis, positive stress tests, or hospital discharge diagnoses as outcomes, which may not allow for preventive measures before significant disease manifests. ^13, 14, 25^ Using ICD-10 codes to diagnose ischemic heart disease, Varbo et al. found a causal risk ratio of 2.8 for ischemic heart disease for a 1-mmol/L (39-mg/dL) increase in RC, which was notably higher than the risk ratio for LDL-cholesterol. ^26^ Kugiyama et al found that higher RC independently predicted development of coronary events in a sample of 135 patients with baseline coronary artery disease, though notably measured RC directly, in comparison to our calculated variable. ^14^

Despite South Asians experiencing a high burden of ASCVD, these populations have not been well represented in the clinical research literature. Prior studies estimate a 1.7- to 4-fold higher risk of ASCVD among South Asian individuals compared to other racial/ethnic groups. ^4, 9, 27^ A recent United Kingdom Biobank analysis by Patel et al. of a large cohort of South Asians and those of European ancestry without cardiovascular disease at baseline demonstrated that over a median follow up of 11 years, South Asians had more than twice the risk of ASCVD events (hazard ratio 2.03) despite nearly identical American Heart Association/American College of Cardiology Pooled Cohort Equations and QRISK3 equations. ^9^ After serial adjustment for several social, behavioral, and clinical factors, there still remained a 43% higher risk of ASCVD among South Asians compared to White Europeans in this analysis.

Despite the promising nature of RC as a potential biomarker, several barriers exist to implementing RC as a risk stratification tool. First, calculated RC with clinically available lipoprotein values and directly measured RC by ultracentrifugation or magnetic resonance-measured only correlate weakly. Studies utilize various definitions of RC ranging from direct measure of IDL and VLDL to triglyceride-based measurements to calculations such as ours. ^13, 28^ Second, while there is extensive literature exploring the effect of various LDL-cholesterol cut-points on atherosclerotic disease development and progression, it is not well understood at which level of RC patients are at risk. One recent retrospective study in North India noted a relatively low RC level among its participants (median 17.0 mg/dL, IQR 12.0–24.0 mg/dL, with only 11.9% of subjects having values > 30 mg/dL). More data are needed to determine the burden of RC in various groups. ^29^

Lastly, there is no agreed-upon definition for CAC progression in the literature. In our sample, 291 individuals had progression of CAC with mean CAC progression 214.5 ± 298.4 Agatston units. The use of CAC as a surrogate for subclinical atherosclerosis may not accurately represent the actual risk that RC poses in development of ASCVD.

## Future directions

There is emerging literature on gene mutations that specifically affect RC levels, including *APOA5* and *LPL*, as established in Nordestgaaard et al. ^30^ Additional Mendelian studies have suggested a prominent role of apoCIII activity in atherosclerotic disease, and development of an antisense oligonucleotide inhibitor of apoCIII is underway. ^31^ In our sample, there was a statistically significant difference in HOMA-IR, HOMA-B, adiponectin, and insulin levels between RC quartiles at baseline; future literature should explore the relationship between RC and these biomarkers.

In summary, in MASALA, higher levels of RC conferred increased odds of incident CAC, independent of traditional ASCVD risk factors, and even after adjusting for behavioral measures, adiposity measures, and advanced metabolic measures. RC may serve as a promising therapeutic target to address residual ASCVD risk.

## Figures and Tables

**Table 1.** Characteristics of MASALA study participants at baseline stratified by remnant cholesterol quartiles.

	All (n = 892)	Q1 (n = 214)	Q2 (n = 226)	Q3 (n = 210)	Q4 (n = 242)	*P*-value

Age (y)	55.3 ± 9.4	55.9 ± 10.4	55.9 ± 9.3	55.4 ± 9.0	54.3 ± 8.7	.209
Men	476 (53.4)	101 (47.2)	107 (47.3)	109 (51.9)	159 (65.7)	<.001*
Income category <$40K	113 (13.0)	28 (13.5)	20 (9.1)	32 (15.7)	33 (13.9)	.799
$40–75k	117 (13.5)	30 (14.5)	29 (13.2)	25 (12.3)	33 (13.9)	
$75–100k	88 (10.1)	19 (9.2)	25 (11.4)	16 (7.8)	28 (11.8)	
>$100k	549 (63.3)	130 (62.8)	145 (66.2)	131 (64.2)	143 (60.3)	
Years lived in United States	27 ± 11	28 ± 11	28 ± 11	27 ± 11	26 ± 10	.194
Education ≥ Bachelor’s degree	783 (87.8)	194 (90.7)	198 (87.6)	185 (88.1)	206 (85.1)	.358*
Systolic blood pressure, mm Hg	125 ± 16	124 ± 17	123 ± 16	126 ± 17	126 ± 13	.21
Diastolic blood pressure, mm Hg	74 ± 10	72 ± 10	73 ± 10	74 ± 10	75 ± 9	.008*
BMI, kg/m^2^	25.9 ± 4.0	24.7 ± 3.9	25.9 ± 4.5	26.4 ± 3.8	26.5 ± 3.6	<.001*
Waist circumference, cm	92.7 ± 10.3	89.4 ± 10.4	92.1 ± 10.9	93.8 ± 9.9	95.2 ± 8.9	<.001*
Hip girth, cm	102.9 ± 8.4	101.2 ± 8.3	102.7 ± 9.1	103.9 ± 8.1	103.6 ± 7.9	.004*
Never smoker	738 (82.7)	183 (85.5)	195 (86.3)	176 (83.8)	184 (76.0)	.003*
Current or former smoker	154 (17.3)	31 (14.5)	31 (13.7)	34 (16.2)	58 (24.0)	
Alcohol use ≥1 drink/wk	295 (33.1)	82 (38.3)	68 (30.1)	60 (28.6)	85 (35.1)	.115
Exercise, MET-min/wk	945 (315–1845)	1140 (420–2205)	945 (315–1755)	840 (248–1680)	833 (368–1650)	<.001*
Total energy, kcals/d	1681 ± 502	1647 ± 502	1681 ± 509	1702 ± 511	1694 ± 488	.683
Hypertension^a^	358 (40.1)	82 (38.3)	86 (38.1)	89 (42.4)	101 (41.7)	.706
No diabetes	668 (74.9)	173 (80.8)	178 (78.8)	154 (73.3)	163 (67.4)	<.001*
Diabetes mellitus^b^	224 (25.1)	41 (19.2)	48 (21.2)	56 (26.7)	79 (32.6)	
Statin use	243 (27.2)	49 (22.9)	66 (29.2)	60 (28.6)	68 (28.1)	.433
Any cholesterol medication use^c^	264 (29.6)	51 (23.8)	72 (31.9)	64 (30.5)	77 (31.8)	.205
Remnant cholesterol, mg/dL	25.8 ± 11.8	13.4 ± 2.7	20.4 ± 1.8	26.9 ± 2.0	41.0 ± 10.3	<.001*
Total cholesterol, mg/dL	188 ± 37	178 ± 32	179 ± 34	192 ± 39	200 ± 36	<.001*
HDL-cholesterol, mg/dL	50 ± 13	59 ± 14	52 ± 13	48 ± 10	43 ± 10	<.001*
LDL-cholesterol, mg/dL	111 ± 32	105 ± 29	107 ± 29	117 ± 36	117 ± 32	<.001*
Triglycerides, mg/dL	128 ± 57	68 ± 14	102 ± 9	134 ± 10	201 ± 49	<.001*
Adiponectin, ng/dL	10,665 (7036–15,344)	14,505 (10,376–19,449)	11,494 (7652–15,379)	10,026 (6677–14,635)	8829 (6098–11,383)	<.001*
Lp(a), mg/dL	17 (9–33)	17 (9–34)	18 (9–33)	17 (9–31)	17 (9–34)	.905
CRP, ug/mL	1.2 (0.6–2.8)	0.9 (0.5–2.4)	1.2 (0.6–2.7)	1.3 (0.7–2.8)	1.5 (0.7–3.2)	.951
Insulin, pmol/L	60.1 (42.0–88.0)	41.8 (29.4–59.0)	57.6 (42.9–82.0)	63.8 (49.0–98.0)	75.0 (54.9–109.0)	<.001*
Resistin, pg/dL	19,920 (16,260–25,010)	19,310 (15,710–24,550)	19,585 (16,030–24,765)	20,010 (16,630–24,190)	20,760 (16,835–25,865)	.837
HOMA-B	103.1 (70.4–151.8)	79.0 (56.0–118.9)	103.6 (71.5–149.3)	113.7 (83.4–164.7)	119.8 (74.1–167.4)	<.001*
HOMA-IR	2.48 (1.62–3.83)	1.60 (1.10–2.56)	2.35 (1.67–3.33)	2.71 (1.92–4.27)	3.27 (2.28–5.00)	<.001*
Fatty liver (HU<40)	82 (9.3)	9 (4.2)	16 (7.1)	20 (9.7)	37 (15.4)	<.001*
Pericardial fat (cm^2^)	52.0 (38.0–73.3)	42.5 (28.0–61.7)	50.4 (36.7–69.7)	56.4 (40.3–76.2)	58.2 (44.0–81.3)	<.001*
Visceral fat area (cm^2^)	126.1 (94.6–165.8)	102.9 (71.0–138.6)	118.4 (91.7–154.5)	135.9 (103.7–172.8)	142.1 (111.7–184.1)	<.001*
Muscle fat area (cm^2^)	21.4 ± 8.7	20.2 ± 9.3	21.5 ± 8.4	22.0 ± 9.2	21.8 ± 7.9	.135
CAC 0	514 (58.1)	126 (59.2)	131 (58.5)	123 (59.1)	134 (55.8)	.434
CAC 1–99	205 (23.2)	44 (20.7)	51 (22.8)	48 (23.1)	62 (25.8)	
CAC ≥100	166 (18.8)	43 (20.2)	42 (18.8)	37 (17.8)	44 (18.3)	

Abbreviations: BMI, body mass index; CAC, coronary artery calcium; CRP, C-reactive protein; HDL, high-density lipoprotein; HOMA-B, homeostasis model assessment of beta-cell function; HOMA-IR, homeostasis model assessment of insulin resistance; HU, Hounsfield Units; LDL, low-density lipoprotein; Lp(a), lipoprotein(a); MET, metabolic equivalent of task.

* The asterisk denotes the values that are statistically significant *P* < .05.

a Hypertension was defined as systolic blood pressure ≥140 mm Hg and/or diastolic blood pressure ≥90 mm Hg and/or use of any antihypertension medication.

b Diabetes mellitus was defined as fasting glucose ≥126 mg/dL and/or use of an antidiabetes mellitus medication.

c Cholesterol medication defined as statin, fibrate, niacin, ezetimibe, or colesevelam.

**Table 2.** Sequential modeling of incident CAC with ASCVD, behavioral, adiposity, and metabolic risk factors, MASALA study.

	OR (95% CI)	*P*-value	OR (95% CI)	*P*-value	OR (95% CI)	*P*-value	OR (95% CI)	*P*-value

Q2 vs Q1	1.68 (0.75–3.77)	.206	1.68 (0.75–3.77)	.2122	1.94 (0.84–4.47)	.1204	1.87 (0.80–4.38)	.1505
Q3 vs Q1	2.24 (1.01–4.98)	.047*	2.27 (1.02–5.07)	.0454*	2.60 (1.13–5.98)	.025*	2.55 (1.09–5.96)	.0311*
Q4 vs Q1	2.57 (1.20–5.51)	.015*	2.57 (1.19–5.53)	.0158*	2.81 (1.26–6.30)	.012*	2.85 (1.24–6.57)	.0141*
Age (y)	1.07 (1.04–1.11)	<.001*	1.07 (1.04–1.11)	<.0001*	1.08 (1.04–1.12)	<.0001*	1.07 (1.04–1.11)	<.0001*
Gender (reference, male)	0.26 (0.15–0.46)	<.001*	0.30 (0.17–0.52)	<.0001*	0.22 (0.12–0.41)	<.0001*	0.30 (0.17–0.55)	<.0001*
Diabetes	1.21 (0.63–2.31)	.563	1.28 (0.66–2.46)	.4679	1.38 (0.69–2.77)	.3662	1.36 (0.63–2.91)	.4332
Smoking (former/current)	1.51 (0.75–3.04)	.253	1.22 (0.59–2.54)	.5882	1.67 (0.81–3.43)	.1618	1.54 (0.75–3.19)	.2418
Cholesterol medication use	1.84 (1.04–3.26)	.038*	1.72 (0.96–3.07)	.0679*	1.74 (0.98–3.11)	.0591*	2.10 (1.15–3.83)	.0156*
Hypertension	2.55 (1.48–4.38)	.001*	2.54 (1.47–4.39)	.0009*	2.55 (1.46–4.45)	.001*	2.61 (1.47–4.61)	.001*
Exercise (per MET-min/wk)			1.00 (1.00–1.00)	.7544				
Alcohol (1+ drink/wk)			1.82 (1.05–3.15)	.0326*				
Visceral fat area (cm^2^)					1.00 (0.99–1.00)	.3559		
Liver attenuation (HU)					1.00 (0.97–1.03)	.9405		
Pericardial fat volume (cm^3^)					1.00 (0.99–1.01)	.7555		
BMI (kg/m^2^)					1.03 (0.95–1.12)	.5011		
HOMA-B							1.00 (1.00–1.01)	.4845
HOMA-IR							0.90 (0.77–1.06)	.2112
Adiponectin (mg/dL)							1.00 (1.00–1.00)	.2325

Abbreviations: ASCVD, atherosclerotic cardiovascular disease; BMI, body mass index; CAC, coronary artery calcium; HOMA-B, homeostasis model assessment of beta-cell function; HOMA-IR, homeostasis model assessment of insulin resistance; HU, Hounsfield Units; MASALA, Mediators of Atherosclerosis in South Asians Living in America; MET, metabolic equivalent of task; OR, odds ratio.

* The asterisk denotes the values that are statistically significant *P* < .10.
